# Supplementary figures and images for: Impact of social distancing on the spread of common respiratory viruses during the coronavirus disease outbreak
Source: PLoS One. 2021 Jun 14;16(6):e0252963. doi: 10.1371/journal.pone.0252963 (PMC8202938; doi:10.1371/journal.pone.0252963)

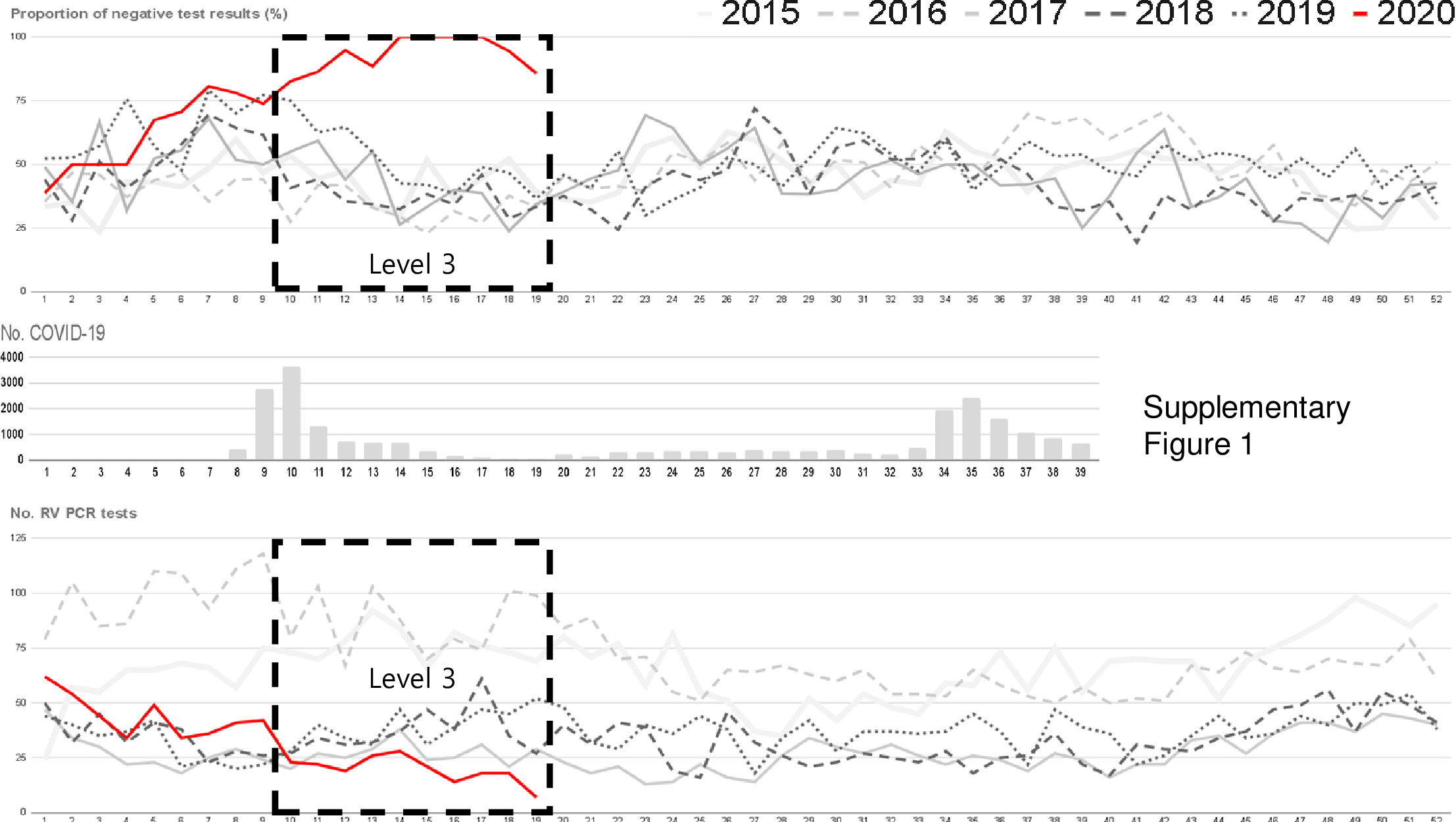

Supplement: S1 Fig — Middle graph shows the weekly number of COVID-19 patients between the 1st week and 39th week of 2020 in South Korea. PCR, Polymerase chain reaction; COVID-19, Coronavirus disease 2019. (TIF) [file pone.0252963.s001.tif]

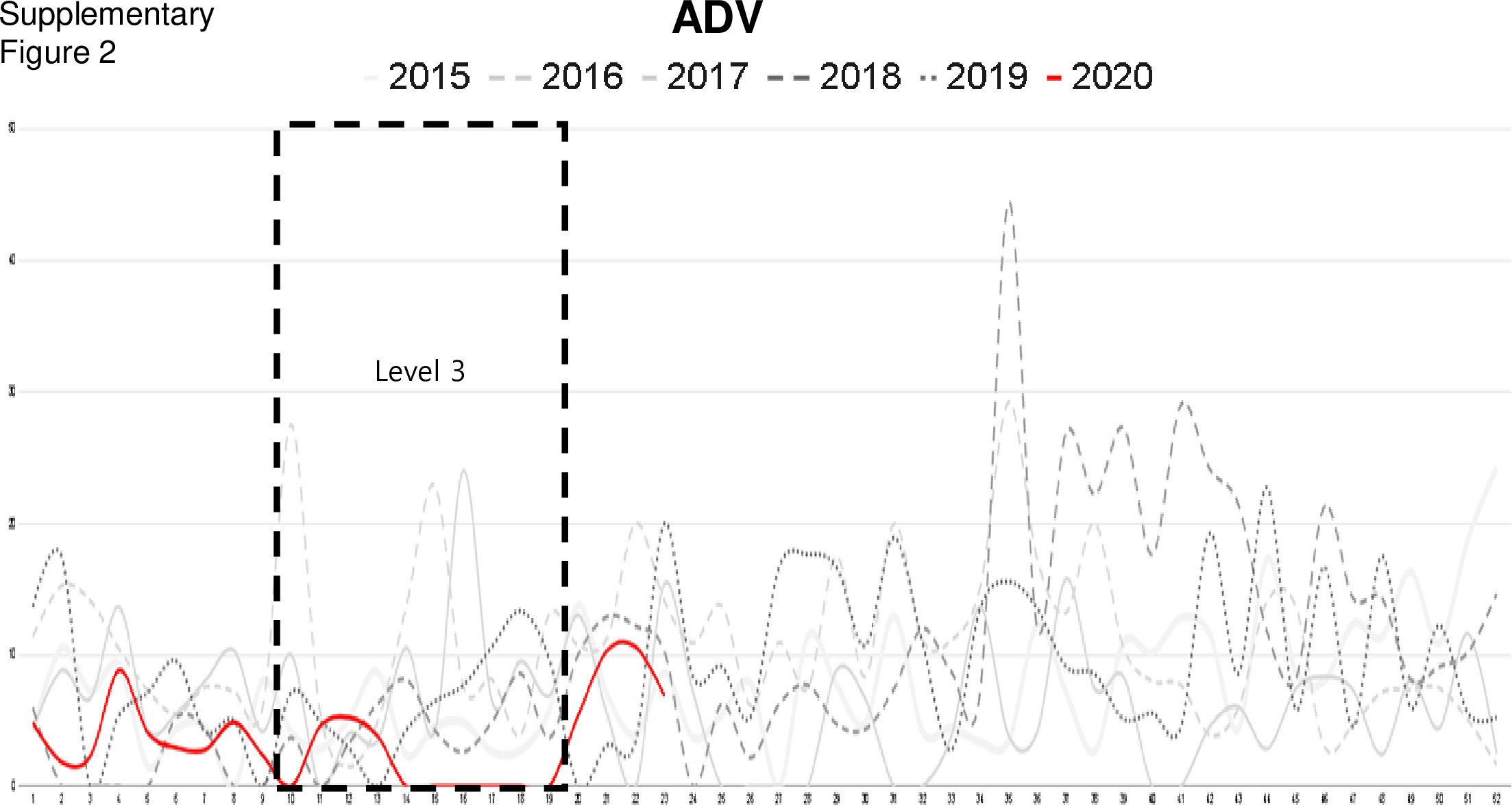

Supplement: S2 Fig — (TIF) [file pone.0252963.s002.tif]

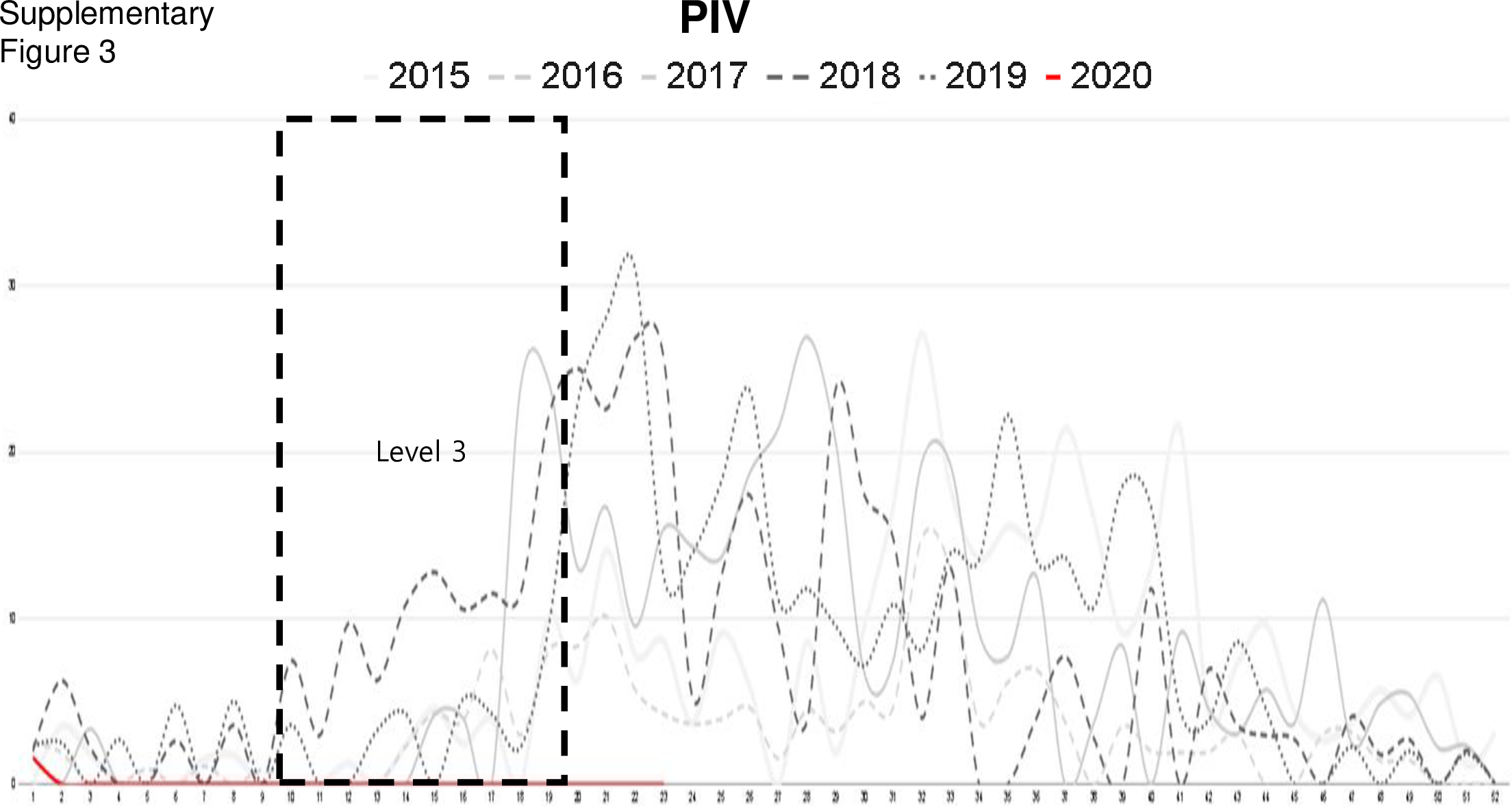

Supplement: S3 Fig — (TIF) [file pone.0252963.s003.tif]

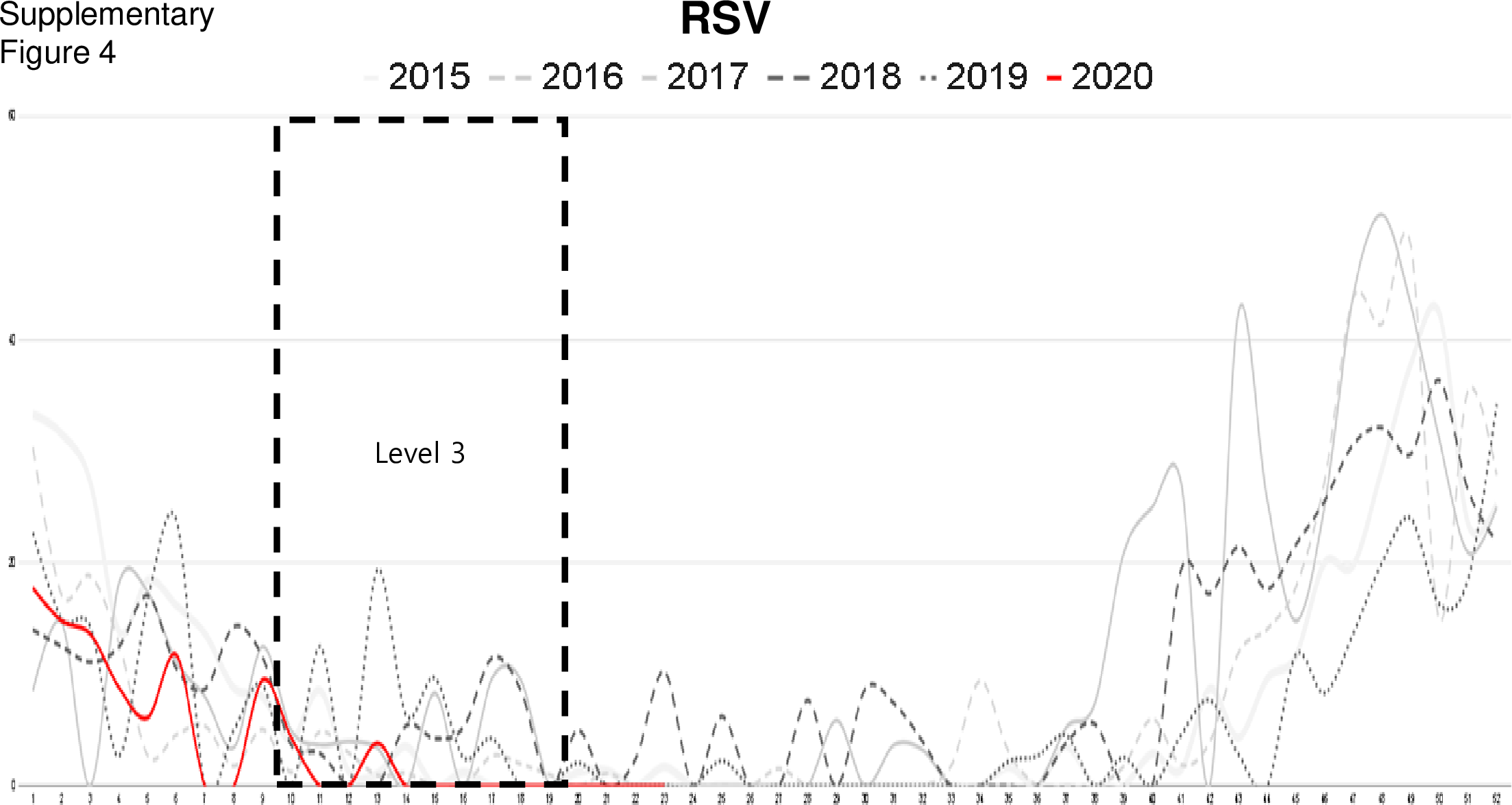

Supplement: S4 Fig — (TIF) [file pone.0252963.s004.tif]

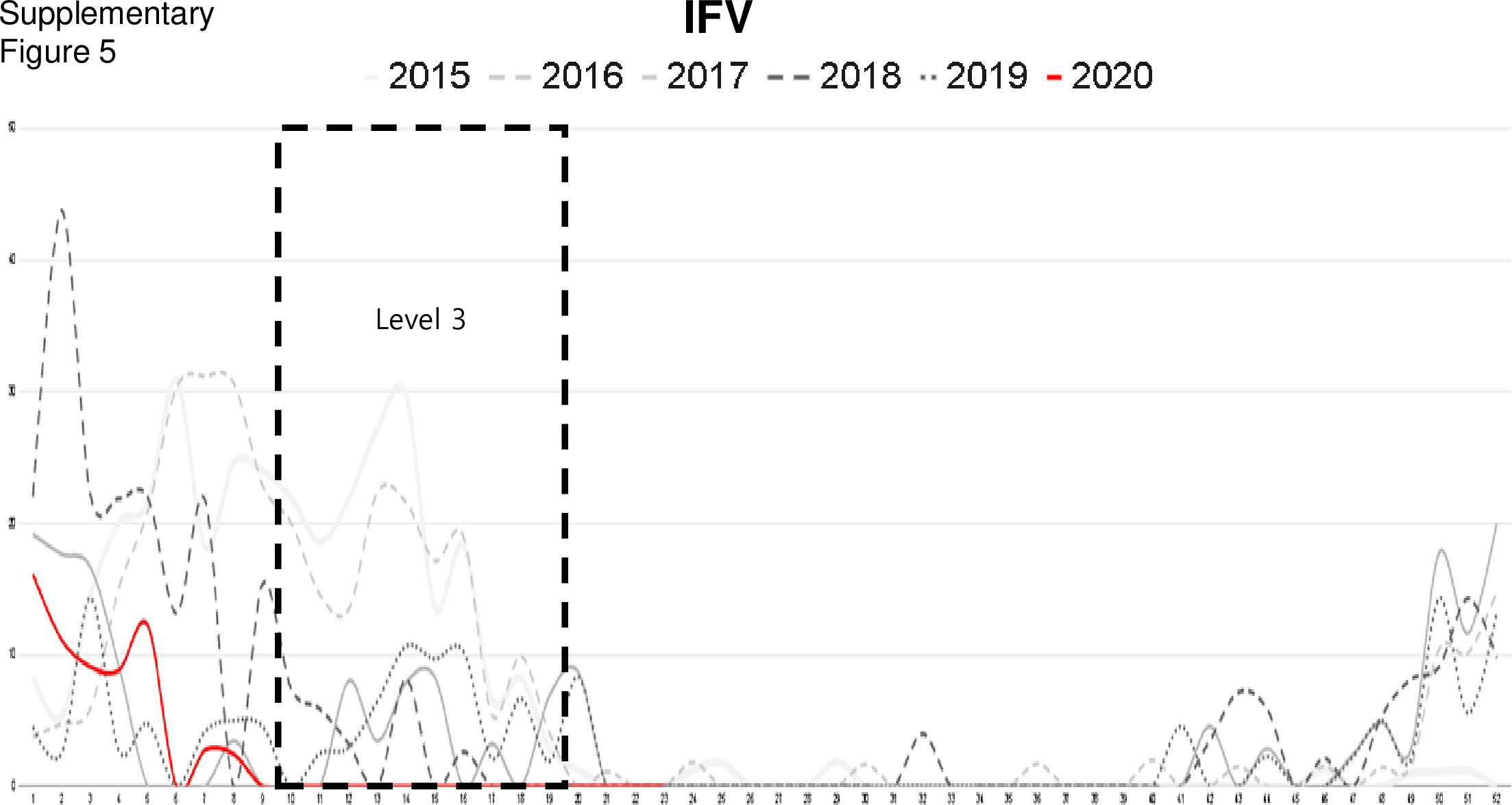

Supplement: S5 Fig — (TIF) [file pone.0252963.s005.tif]

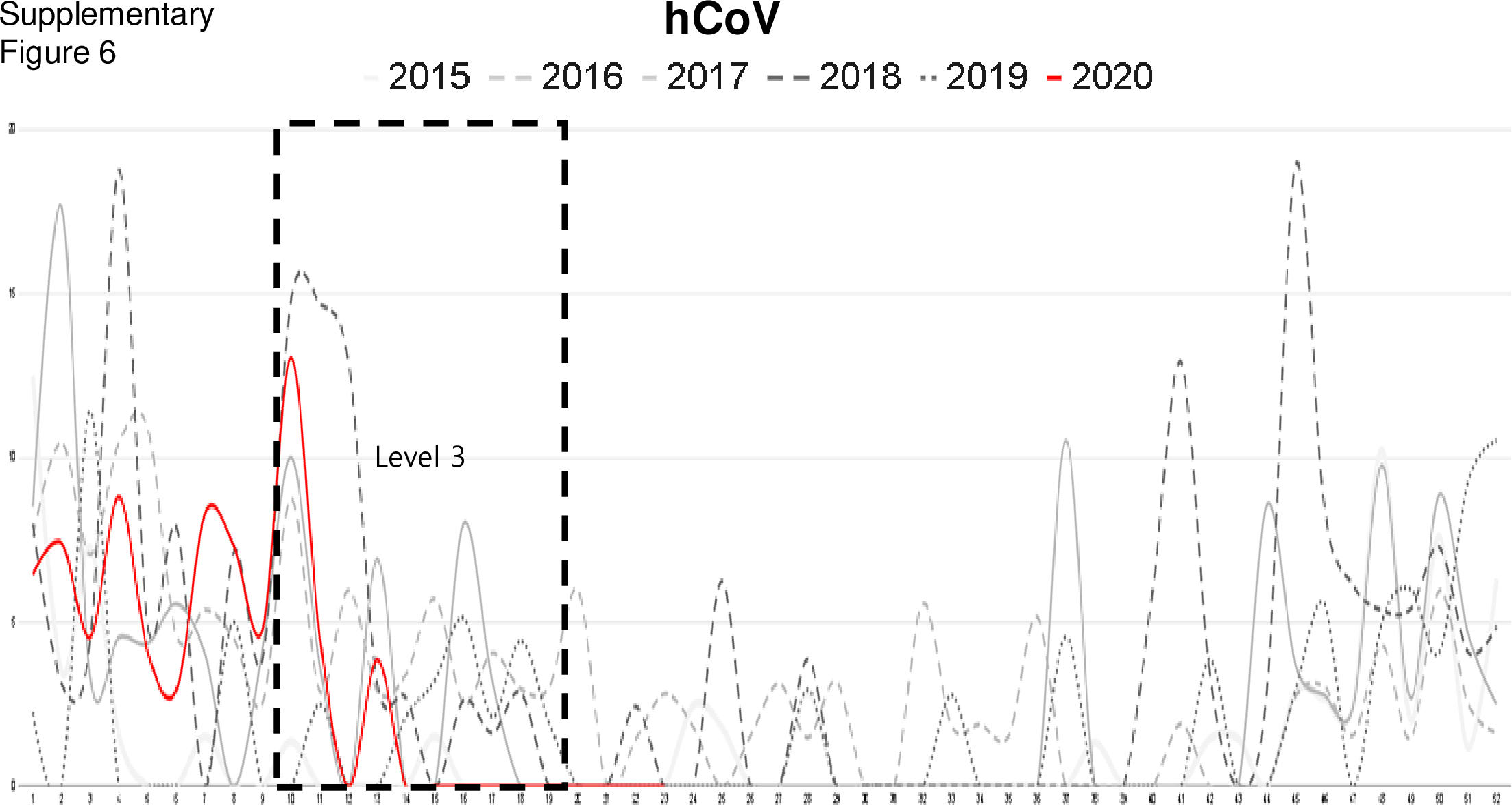

Supplement: S6 Fig — (TIF) [file pone.0252963.s006.tif]

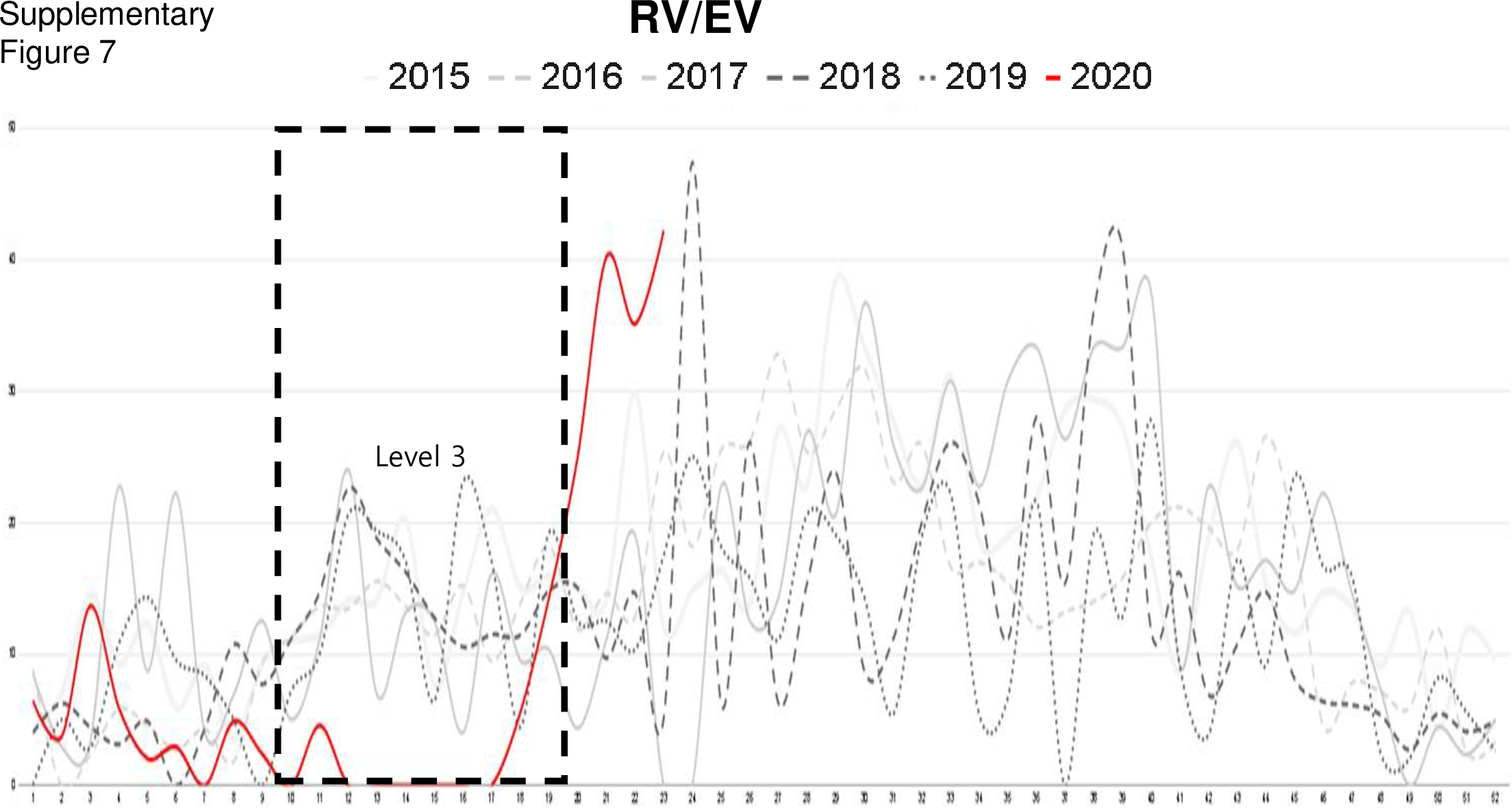

Supplement: S7 Fig — (TIF) [file pone.0252963.s007.tif]

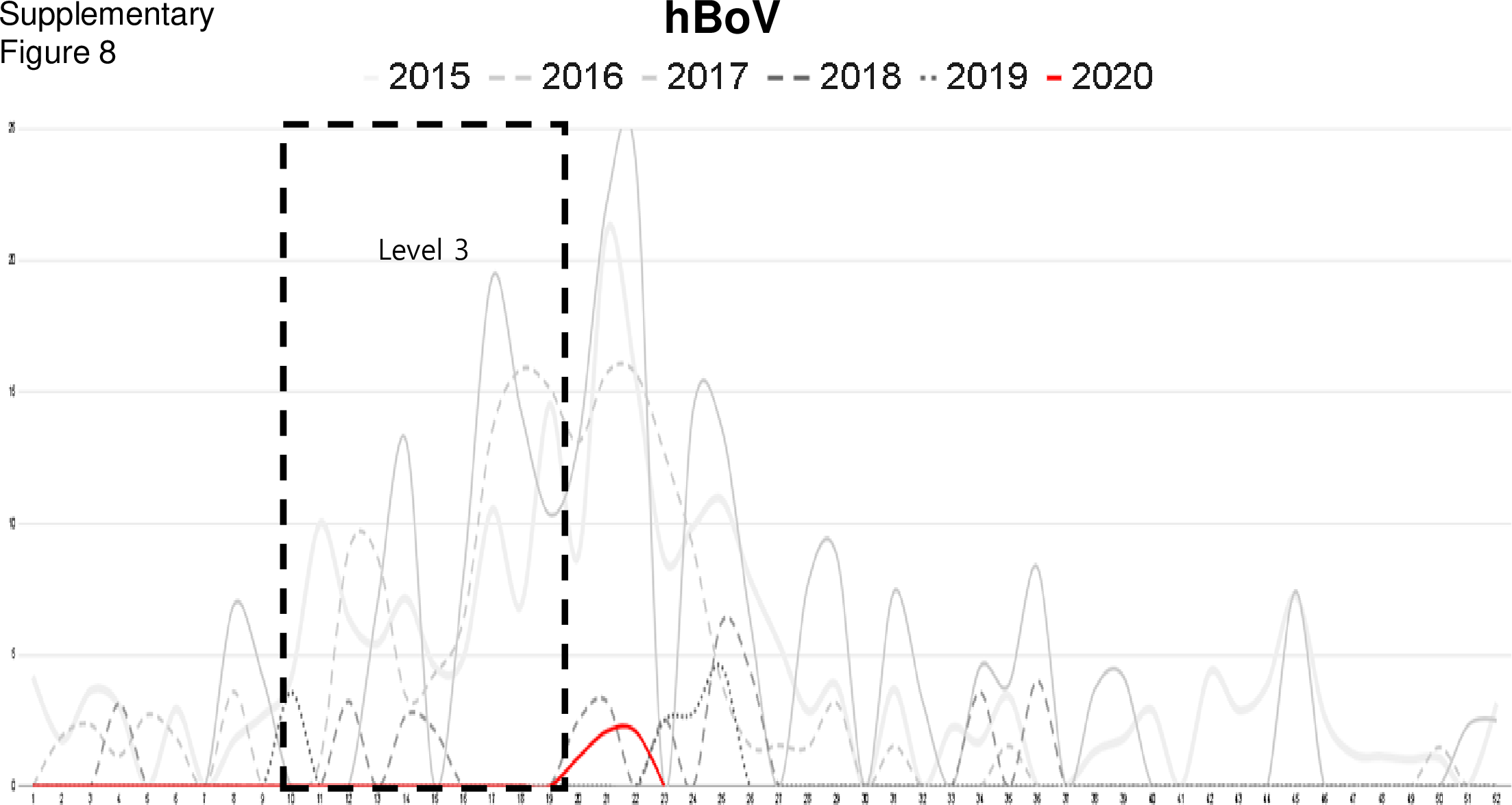

Supplement: S8 Fig — (TIF) [file pone.0252963.s008.tif]

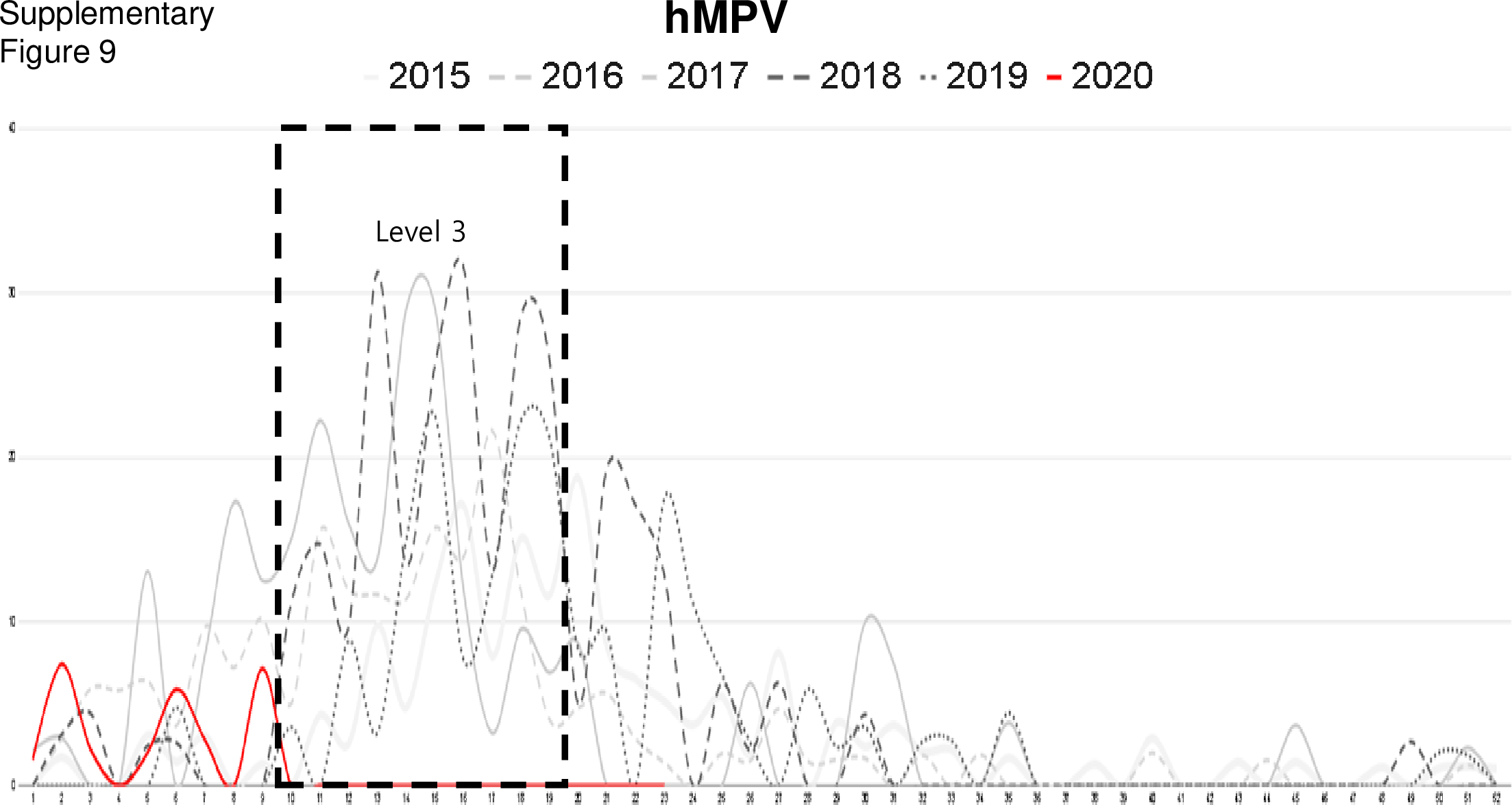

Supplement: S9 Fig — (TIF) [file pone.0252963.s009.tif]
