# Supplementary material for: Impact of social distancing on the spread of common respiratory viruses during the coronavirus disease outbreak
Source: PLoS One. 2021 Jun 14;16(6):e0252963. doi: 10.1371/journal.pone.0252963 (PMC8202938; doi:10.1371/journal.pone.0252963)
Supplement: Attachment — Submitted filename: RESPONSE TO REVIEWER.docx [file pone.0252963.s010.docx]

**RESPONSE TO REVIEWER’S COMMENTS**

**5. Review Comments to the Author

Please use the space provided to explain your answers to the questions above. You may also include additional comments for the author, including concerns about dual publication, research ethics, or publication ethics. (Please upload your review as an attachment if it exceeds 20,000 characters)**

**Reviewer #1: In this manuscript, Kim and colleagues determined the effects of social distancing on the transmission of eight common respiratory viruses in Korea. Using the data from Korean Center for Disease Control and Prevention and a local University teaching hospital, the authors found an increased negative PCR rate for common respiratory viruses, which was correlated with the levels of practiced social distancing. The authors also examined PCR positive rates for selected respiratory viruses. The rate for parainfluenza virus, influenza virus, human coronavirus, human metapneumovirus was significantly reduced. No significant changes were seen for adenovirus and bocavirus. The positive rate for human rhinovirus/enterovirus was reduced in level 3 but surprisingly increased during the level 1 social distancing.
Overall, this manuscript provides new information which could help us understand the effects of social distancing on the transmission of respiratory viruses. My concerns and comments are listed as following:

Major comments:
1. It is interesting that no significant changes were seen for adenovirus and bocavirus, and the positive rate for human rhinovirus/enterovirus was reduced in level 3 but surprisingly increased in level 1. However, the positive rate itself is not sufficient to interpret and understand the data. The authors should add the number of positive PCR detection in addition to the positive rate in Figure 2 for each virus.**

*Answer) Following your instructions, we made new figures of positive PCR detection for eight respiratory viruses. Also, we added some descriptions to help interpret and understand the revised data.*

*New figures) Figure 3*

*Added descriptions for the figures) Results*

Weekly numbers of each of eight respiratory viruses during the periods of social distancing were generally lower than those without social distancing (Fig 3), because less than half of the respiratory virus PCR tests were conducted during the former periods compared to the latter periods. Only in the case of RV/EV during the period of level 1 social distancing, the weekly numbers of RV/EV were similar to those without social distancing [Fig 3 (F-2)], because of much higher positive rates during the level 1 social distancing period [Fig 3 (F-1)], despite the small number of respiratory virus PCR tests (Fig 1).

**Besides, which age group has the increased human rhinovirus/enterovirus rate?**

*Answer) When K-CDC announced data on respiratory viruses on its web page, it did not disclose detailed data classified by age. Prior to this study, we had requested that age-specific data be shared with us, but we did not get the K-CDC’s cooperation. Unfortunately, as the COVID-19 pandemic came, it was more difficult to obtain such data, and we cannot provide additional data at this time. If the data will be available later, it is considered to be a part that must be analyzed.*

**2. The figures 2 and 3 are placed in the wrong order and didn’t the match the citations in the main manuscript.**

*Answer) We corrected the figure order problem you pointed out. Also, a table that was not mentioned in the text in the previous our manuscript was also inserted in the text as table 2.*

**3. Page 15, lines 159-169. The authors wrote: “PCR tests for SARS-CoV-2 were performed in
2,426,535 individuals between the 7th and 42nd weeks of 2020 in South Korea (an average of
67,404 tests per week). Why the number of the weekly average test ranged from 100-300 according to Figure 1. The authors need to explain this.

Also, it is not clear if all listed respiratory viruses were examined in addition to SARS-CoV2 for the same patient.**

*Answer) The problem is that SARS-CoV-2 PCR and respiratory virus PCR were performed on different patient groups. Since this part was not clearly presented in the results section, causing misunderstandings. The results section was changed to the following.*

*Changed texts) results section*

The results of the following three PCR tests performed on different patient groups were used in this study – 1) respiratory virus PCR test results were reported by the K-CDC for 303 weeks, but data on the number of performed tests were only available for 176 weeks (35,185 tests, an average of 200 tests per week). 2) respiratory virus PCR tests were performed in 13,045 individuals at the large university hospital in 280 weeks (an average of 47 tests per week). 3) PCR tests for SARS-CoV-2 were performed in 2,426,535 individuals between the 7th and 42nd weeks of 2020 in South Korea (an average of 67,404 tests per week), and 25,129 individuals were diagnosed with COVID-19.

*We guess you will know why the average of 100-300 tests per week was recorded.*

**4. The authors may want to add a short summary about the features of the transmission of common respiratory viruses in the introduction section. For instance, when does rhinovirus/enterovirus season occur in Korea?**

*Answer) Following your instruction, we changed the manuscript like the followings.*

*Changed texts) Introduction*

Of these measures, social distancing is considered to help prevent the spread of various infectious diseases, including influenza and COVID-19, since it fundamentally reduces contact among people [4, 5]. ~~Although social distancing has been reported in the past and during the SARS epidemic [6], it is probably the first time in this century that social distancing has been widely applied across the country.~~ To prepare for future pandemics caused by various pathogens, it is necessary to evaluate the impact of national social distancing not only on the spread of COVID-19, but also on the transmission of various infectious diseases~~, including COVID-19~~. In particular, the impact on common respiratory virus infections that may be the cause of ongoing pandemic needs to be examined further. Like other countries in the northern hemisphere, the following eight common respiratory viruses circulate in Korea. Influenza virus (IFV), respiratory syncytial virus (RSV), and human coronavirus (~~h~~C~~o~~OV) are prevalent in the cold season, human metapneumovirus (~~h~~MPV) and human bocavirus (~~h~~B~~o~~OV) are in spring, and parainfluenza virus (PIV) is in summer. Rhinovirus (RV) shows bimodal peaks that increase in spring and fall, but like adenovirus (ADV), it circulates in all seasons [6, 7]. Therefore, we aimed to investigate the impact of social distancing on the spread of these eight common respiratory virus~~es~~ ~~(RV)~~ infections by examining the changes in the rates of these infections before and after the implementation of social distancing for the COVID-19 outbreak.

**Minor comments:

1. Rather than using RV throughout the manuscript, please spell it out and use respiratory viruses instead. It may confuse the reader as RV often refers to rhinovirus.**

*Answer) Following your instructions,* ***RV*** *was spelled out as* ***respiratory virus*** *throughout the manuscript, so as not be confused with rhinovirus (RV)*

**2. Page 11, lines 93-95. Please consider removing this sentence.**

*Answer) following your instructions, we removed the sentence.*

*Changed texts) Introduction*

Of these measures, social distancing is considered to help prevent the spread of various infectious diseases, including influenza and COVID-19, since it fundamentally reduces contact among people [4, 5]. ~~Although social distancing has been reported in the past and during the SARS epidemic [6], it is probably the first time in this century that social distancing has been widely applied across the country.~~

**3. Is the study hospital the same as the teaching hospital?**

*Answer) In order not to be confused in this part, the following changes have been made.*

*Changed texts) Results*

The results of the following three PCR tests performed on different patient groups were used in this study – 1)

respiratory virus PCR test results were reported by the K-CDC for 303 weeks, but data on the number of performed tests were only available for 176 weeks (35,185 tests, an average of 200 tests per week). 2) respiratory virus PCR tests were performed in 13,045 individuals at the ~~study~~large university hospital in 280 weeks (an average of 47 tests per week). 3) PCR tests for SARS-CoV-2 were performed in 2,426,535 individuals between the 7th and 42nd weeks of 2020 in South Korea (an average of 67,404 tests per week), and 25,129 individuals were diagnosed with COVID-19.

**4. Page 9, lines 164-165. This sentence is confusing. Since the reason was given in the discussion section (page 22), the authors may want to simply state that the PCR test number decreased from the beginning of COVID.**

*Answer) In order not to be confused in this part, the following changes have been made.*

*Changed texts) Results*

Figure 1 presents the curves of the weekly number of respiratory virus PCR tests and the weekly proportion of negative test results in the K-CDC dataset, with the graph of the weekly number of patients with COVID-19, during the study period. ~~As the number of COVID-19 patients surged from the 9th week of 2020, t~~The number of ~~RV~~respiratory virus PCR tests rapidly decreased from the 9th week of 2020, and from the 10th week to the 42nd week of 2020, the number of respiratory virus PCR tests continued to be ≤100. The proportion of negative test results reached 87.8% in the 12th week of 2020, exceeded 75% during level 3 of social distancing, and decreased by the 19th week.

**Thank you for your thoughtful comments !**
