# Supplementary material for: Impact of social distancing on the spread of common respiratory viruses during the coronavirus disease outbreak
Source: PLoS One. 2021 Jun 14;16(6):e0252963. doi: 10.1371/journal.pone.0252963 (PMC8202938; doi:10.1371/journal.pone.0252963)
Supplement: Attachment — Submitted filename: RESPONSE TO REVIEWER_2021_05_14.docx [file pone.0252963.s011.docx]

**RESPONSE TO REVIEWER’S COMMENTS**

**Additional Editor Comments:**

**The manuscript describes the changes in the detection rate of the common respiratory virus infections by a retrospective study during COVID-19 pandemic in South Korea.**

**Methods>Data collection:**

**Line 97: Characteristics of the K-CDC data have been described elsewhere. As this is an important piece of information for the reader to understand the nature of such, a brief description would be essential for easy reference.**

*Answer) Following your instruction, I revised the manuscript*

*From)*

The national respiratory virus surveillance dataset provided by the K-CDC during the study period was retrieved from the K-CDC website [8]. The characteristics of the K-CDC data have been described elsewhere [6].

*To)*

The national respiratory virus surveillance dataset made from Korea Influenza and Respiratory Surveillance System (KINRESS) organized by the K-CDC was retrieved from the K-CDC website [8]. The KINRESS is the public source of laboratory-based epidemiological data showing community outbreaks of influenza and other common respiratory viruses in Korea. Thirty-six primary or secondary clinics throughout the country (recently increased to 52) are providing the K-CDC with respiratory specimens of patients who visit the clinics for acute respiratory illness [6].

**Result:**

**Line 170: Figure 2, are these data points from 2020 only or all years? How about the resolution of the data, is one dot representing the % of a week?**

*Answer) One hollow circle in the figure represents one week during the study period. How many weeks have been included in the figure is described in detail in the figure legends.*

*From)*

**Fig 2. Mean proportion of negative respiratory virus PCR test results and the mean number of respiratory virus tests according to the level of social distancing**

*To)*

**Fig 2. Mean proportion of negative respiratory virus PCR test results according to the level of social distancing (between the 1st week of 2015 and the 42nd week of 2020; a total of 303 weeks) and the mean number of respiratory virus tests according to the level of social distancing (between the 23rd week of 2017 and the 39th week of 2020; a total of 176 weeks). The horizontal line represents the mean value, and the hollow circle represents the value for each week.**

**Table 2. Mann-Whitney U test is for the comparison between two groups, do the authors mean the Kruskal-Wallis test? Please also check the proper post hoc test to be applied.**

*Answer) Table 2 is the result of statistical analysis of the data shown in Fig 2. Since there are 4 groups in Fig 2, the overall trend was confirmed through the Kruskal-Wallis test, and the results are shown not in the Table 2, but in Lines 172–173.*

*Line 172-173)*

The more stringent the level of social distancing, the higher was the proportion of negative respiratory virus PCR test results (*P* < 0.001 using the Kruskal-Wallis test…

*Answer) If the data of Fig 2 with 4 groups are compared with each other by 2 groups, a total of 6 comparisons are made. Since the comparison is between 2 groups, the Mann-Whitney U test was used – you can see the 6 comparisons in Table 2. I revised the manuscript to clarify this point.*

*From)*

Figure 2 shows the mean proportion of negative respiratory virus PCR test results and the mean number of respiratory virus PCR tests according to each level of social distancing. The more stringent the level of social distancing, the higher was the proportion of negative respiratory virus PCR test results (*P* < 0.001 using the Kruskal-Wallis test; the results of post hoc comparisons are presented in Table 2). The number of respiratory virus PCR tests was significantly different between the periods with and without social distancing (*P* < 0.001 using the Kruskal-Wallis test); however, there were no significant differences according to the level of social distancing in the number of respiratory virus PCR tests.

*To)*

Figure 2 shows the mean proportion of negative respiratory virus PCR test results and the mean number of respiratory virus PCR tests according to each level of social distancing. The more stringent the level of social distancing, the higher was the proportion of negative respiratory virus PCR test results (*P* < 0.001 using the Kruskal-Wallis test; the results of post hoc comparisons using Mann-Whitney *U* test with Bonferroni correction are presented in Table 2). The number of respiratory virus PCR tests was significantly different between the periods with and without social distancing – adjusted *P* value is <0.001 when comparing the “no social distancing group” and the level 1, 2, and 3 social distancing group, respectively (Table 2). However, when the three social distancing groups were compared with each other in the number of respiratory virus PCR tests, there was no significant difference (Table 2).

**In Figure 3, the legend is not clear (number verse proportion). There are a series of (A-1) (A-2), (B-1) (B-2) in which not each of them was mentioned in the main text, the author might consider showing figures which provide the key messages to help the reader’s understanding. Please either clarify the differences in the text and so as to demonstrate why it is essential to show both the actual number and the percentage. In addition, please consider showing only one of them to make the message clearer for the reader). Please state clearly what statistical test was performed.**

*Answer)*

- *I agree that each graph has not been fully explained in the main text, although there are many graphs in Fig 3. First, the legends in Fig 3 were modified as follows.*

*From)*

**Fig 3. Weekly number and positive rates of respiratory viruses in South Korea between the 1st week of 2015 and the 42nd week of 2020**

3A. Adenovirus (ADV); 3B. Parainfluenza virus (PIV); 3C. Respiratory syncytial virus (RSV); 3D. Influenza (IFV); 3E. Human coronavirus (COV); 3F. Rhinovirus/enterovirus (RV/EV); 3G. Human bocavirus (BOV); Figure 3H. Human metapneumovirus (MPV). Each of gray squares represents the periods of level 3, level 1, and level 2 social distancing.

*To)*

**Fig 3. Weekly number and positive rates of respiratory viruses in South Korea between the 1st week of 2015 and the 42nd week of 2020**

A-1. Proportion of Adenovirus (ADV); A-2. Number of ADV; B-1. Proportion of Parainfluenza virus (PIV); B-2. Number of PIV; C-1. Proportion of Respiratory syncytial virus (RSV); C-2. Number of RSV; D-1. Proportion of Influenza (IFV); D-2. Number of IFV; E-1. Proportion of Human coronavirus (COV); E-2. Number of COV; F-1. Proportion of Rhinovirus/enterovirus (RV/EV); F-2. Number of RV/EV; G-1. Proportion of Human bocavirus (BOV); G-2. Number of BOV; H-1. Proportion of Human metapneumovirus (MPV); H-2. Number of MPV. Each of gray squares represents the periods of level 3, level 1, and level 2 social distancing.

*Answer) Since Fig 3 and Table 3 are helpful to each other in understating the results, it has been modified to mention both the main contents of Fig 3 and Table 3 for each virus.*

*From)*

Figure 3 presents the curves of the weekly number/weekly positive rates for each of the eight respiratory viruses. In Table 3, the mean weekly positive rates of respiratory viruses were compared between the social distancing period and the corresponding periods of 2015–2019. Additionally, the same comparisons were conducted according to the three levels of social distancing (Table 3). During the social distancing period, the mean positive rates for PIV, IFV, COV, and MPV were significantly lower than those during the same period in 2015–2019, when social distancing was not implemented (0.1% vs. 9.3%, *P* <0.001; 0.1% vs. 7.2%, *P* <0.001; 0.4% vs. 2.3%, *P* <0.001; and 0.2% vs. 5.3%, *P* <0.001, respectively). This trend was maintained during each level of social distancing. The mean positive rate for RSV during level 2 social distancing was lower than that during the same period in 2015–2019 (0.3% vs. 1.8%, *P* = 0.004). However, considering both Table 3 and Figure 3, social distancing was implemented outside the general RSV outbreak period; thus, it was difficult to observe the effects of social distancing with respect to RSV positivity in this dataset. In the case of ADV and BOV, there were no differences in mean positive rates between the social distancing period and the same period in 2015–2019 (6.2% vs. 6.2%, *P* = 0.906 and 2.6% vs. 3.1%, *P* = 0.432, respectively). In the case of RV/EV, the mean positive rate during level 3 social distancing was lower than that in the same period in 2015–2019 (8.5% vs. 19.0%, *P* <0.001); however, during level 1 social distancing, the mean positive rate was higher than that in the same period in 2015–2019 (38.3% vs. 19.4%, *P* <0.001). During level 2 social distancing, the mean positive rate for RV/EV was not different from that in the same period in 2015–2019, but it was observed that the weekly positive rate for RV/EV decreased in the latter part of this period [Fig 3 (F-1)]. Weekly numbers of each of eight respiratory viruses during the periods of social distancing were generally lower than those without social distancing (Fig 3), because less than half of the respiratory virus PCR tests were conducted during the former periods compared to the latter periods. Only in the case of RV/EV during the period of level 1 social distancing, the weekly numbers of RV/EV were similar to those without social distancing [Fig 3 (F-2)], because of much higher positive rates during the level 1 social distancing period [Fig 3 (F-1)], despite the small number of respiratory virus PCR tests (Fig 1). Graphs of weekly positive rates for the eight respiratory viruses from the university hospital dataset are presented in S2 Fig.

*To)*

Figure 3 presents the curves of the weekly number/weekly positive rates for each of the eight respiratory viruses. In Table 3, the mean weekly positive rates of respiratory viruses were compared between the social distancing period and the corresponding periods of 2015–2019. Additionally, the same comparisons were conducted according to the three levels of social distancing. Each of PIV, IFV, COV, and MPV is rarely observed in the proportion graphs and number graphs in 2020, when social distancing was implemented, unlike 2015-2019 (B-1, B-2, D-1, D-2, E-1, E-2, H-1, and H-2 of Fig 3). In Table 3, the mean positive rates for PIV, IFV, COV, and MPV during the social distancing period were significantly lower than those during the same period in 2015–2019, when social distancing was not implemented (0.1% vs. 9.3%, *P* <0.001; 0.1% vs. 7.2%, *P* <0.001; 0.4% vs. 2.3%, *P* <0.001; and 0.2% vs. 5.3%, *P* <0.001, respectively). This trend was maintained during each level of social distancing (Table 3). The mean positive rate for RSV during level 2 social distancing was lower than that during the same period in 2015–2019 (0.3% vs. 1.8%, *P* = 0.004). However, considering both Table 3 and Figure 3 (C-1 and C-2), social distancing was implemented outside the general RSV outbreak period; thus, it was difficult to observe the effects of social distancing with respect to RSV positivity in this dataset. The number of ADV and BOV each appeared to decrease in 2020 compared to 2015-2019 period in the number graphs (A-2 and G-2 of Fig 3), but there was no significant difference between the 2015-2019 period and 2020 in the proportion graphs (A-1 and G-1 of Fig 3). In Table 3, there were no differences in mean positive rates of ADV and BOV between the social distancing period and the same period in 2015–2019 (6.2% vs. 6.2%, *P* = 0.906 and 2.6% vs. 3.1%, *P* = 0.432, respectively). In the proportion graph, RV/EV decreased during the level 2-3 social distancing period in 2020 compared to 2015-2019, but increased in the level 1 social distancing period compared to 2015-2019 (F-1 of Fig 3). In the number graph of RV/EV, the number decreased during the level 2-3 social distancing period, but increased to a level similar to that of 2015-2019 during the level 1 social distancing period (F-2 of Fig 3). The mean positive rate of RV/EV during level 3 social distancing was lower than that in the same period in 2015–2019 (8.5% vs. 19.0%, *P* <0.001); however, during level 1 social distancing, the mean positive rate was higher than that in the same period in 2015–2019 (38.3% vs. 19.4%, *P* <0.001) (Table 3). Except for RV/EV of the level 1 social distancing period, weekly numbers of each of seven respiratory viruses during the periods of social distancing were generally lower than those without social distancing (Fig 3), because less than half of the respiratory virus PCR tests were conducted during the former periods compared to the latter periods. Graphs of weekly positive rates for the eight respiratory viruses from the university hospital dataset are presented in S2 Fig.

*Answer) The actual number graph and the percentage graph were both presented, and not changed to present only one of them. The reason is that only percentage graphs for each virus were presented at the time of initial submission, but it was revised according to reviewer’s recommendation that the interpretation may be unclear without actual number graph. I think the recommendation is appropriate.*

**Discussion:**

**Paragraph 1, please state the nature of the respiratory samples, were those from cases with respiratory symptoms or random surveillance? What are the demographics of these samples? Is there any change in the proportion of age, or nature of the subjects who provided the sample for the test? Is there a change in the respiratory virus surveillance sample collection criteria during the COVID-19 pandemic? This determines the validity of the study.**

*Answer)*

*As mentioned during the revision of the methods section above, K-CDC’s national surveillance system collected samples of patients who visited primary or secondary clinics in Korea with acute respiratory illness. This system started 20 years ago and reports data every week. So, it is difficult to think that there is a significant change in the characteristics of the sample collection and visiting patients, since the data has been steadily collected from the clinical practice for a long time.*

*Detailed information on demographics of participating patients cannot be obtained in the data. I replied to the previous reviewer that it was difficult to obtain such additional data due to realistic circumstances. Please refer to the previous “response to reviewers”.*

*Lastly, there must have been changes in sample collection since the COVID-19 pandemic began. Please refer to this as I have already described this problem in the 5^th^ and 6^th^ paragraphs in the discussion (reduction of the number of patients and the corresponding decrease in the number of PCR tests).*

**What is special about the level 1 social distancing and therefore the doubled EV/RV rate?**

*Answer) As for the social distancing level, you can see the difference in Table 1, which is summarized in the main text as follows.*

*From)*

The proportion of negative respiratory virus PCR test results increased significantly during the social distancing period in both the K-CDC and hospital datasets. The more stringent the level (levels 3 and 2) of social distancing, the higher was the proportion of negative respiratory virus PCR test results. The weekly positive rates for PIV, IFV, COV, and MPV decreased significantly during the social distancing period. The weekly positive rate of RV/EV also showed a tendency to decrease as the level of social distancing increased, but during level 1 of social distancing, it increased compared to the rate observed in previous years (when social distancing was not performed).

*To)*

The proportion of negative respiratory virus PCR test results increased significantly during the social distancing period in both the K-CDC and hospital datasets. The more stringent the level (levels 3 and 2) of social distancing, the higher was the proportion of negative respiratory virus PCR test results. The weekly positive rates for PIV, IFV, COV, and MPV decreased significantly during the social distancing period. The weekly positive rate of RV/EV also showed a tendency to decrease as the level of social distancing increased, but during level 1 of social distancing, when the intensity of and compliance with social distancing decrease, the rate increased compared to that observed in previous years (when social distancing was not performed).

**Overall:**

**The style of writing is like a laboratory report rather than a manuscript, and at the same time, the legend of the figures did not guide the interpretation of the data clearly.**

*Answer) It seems to be because there are many figures and tables, so I could not effectively organize and explain. Hopefully this modification makes it a little better.*

***Thank you for your thoughtful comments!***
