# Supplementary material for: Impact of social distancing on the spread of common respiratory viruses during the coronavirus disease outbreak
Source: PLoS One. 2021 Jun 14;16(6):e0252963. doi: 10.1371/journal.pone.0252963 (PMC8202938; doi:10.1371/journal.pone.0252963)
Supplement: Attachment — Submitted filename: RESPONSE TO REVIEWER_2021_05_22.docx [file pone.0252963.s012.docx]

**RESPONSE TO REVIEWER’S COMMENTS**

**Additional Editor Comments (if provided):**

**The arrangement of the figure legends (they appeared in the middle of the text), and extra lines e.g. row 173 and 193 make things difficult to understand.**

**The main text is like a report describing the numbers indicated in the tables and figures, but not the usual format of a writing to elaborate research outcome with clear ideas being supported by tables and figure. Authors might consider to restructure the result part.**

*Answer) There are too many figures and tables in the results section, so when I tried to explain the in detail, it became very complicated, and it seems that I failed to show the core contents of this study. To improve this, the contents of the results section have been greatly reduced and only the core sentences have been included. The symbols of respiratory viruses in Fig. 3 were also modified in the order of A, B, and C from the first appearing in the main text.*

*From) The 2^nd^ paragraph of the results*

~~Figure 1 presents the curves of the weekly number of respiratory virus PCR tests and the weekly proportion of negative test results in the K-CDC dataset, with the graph of the weekly number of patients with COVID-19, during the study period~~. The number of respiratory virus PCR tests rapidly decreased from the 9th week of 2020, and from the 10th week to the 42nd week of 2020, the number of respiratory virus PCR tests continued to be ≤100. ~~The proportion of negative test results reached 87.8% in the 12th week of 2020, exceeded 75% during level 3 of social distancing, and decreased by the 19th week.~~ ~~The proportion of negative test results was maintained around 50% during level 1 social distancing but gradually increased with the start of level 2 social distancing; it increased to 86.1% in the 41st week (Figure 1).~~ A decrease in the number of respiratory virus PCR tests and an increase in the proportion of negative test results during level 3 social distancing were also observed in the university hospital dataset (S1 Fig).

**Fig 1. Weekly proportion of negative respiratory virus PCR test results (upper graph), weekly number of COVID-19 patients (middle graph), and weekly number of respiratory virus PCR tests (lower graph) in South Korea** **between the 1st week of 2015 and the 42nd week of 2020.**

PCR, Polymerase chain reaction; COVID-19, Coronavirus disease 2019

*To) The 2^nd^ paragraph of the results*

As the number of COVID-19 infections increased rapidly from the 9^th^ week in 2020 and social distancing was implemented from the 10^th^ week, the number of PCR tests decreased by less than a half compared to 2015-2019, and the respiratory virus PCR results was almost negative (Figure 1). The same changes are observed in the university hospital data (S1 Fig).

**Fig 1. Weekly proportion of negative respiratory virus PCR test results (upper graph), weekly number of COVID-19 patients (middle graph), and weekly number of respiratory virus PCR tests (lower graph) in South Korea between the 1st week of 2015 and the 42nd week of 2020.** PCR, Polymerase chain reaction; COVID-19, Coronavirus disease 2019.

*From) The 3^rd^ paragraph of the results*

~~Figure 2 shows the mean proportion of negative respiratory virus PCR test results and the mean number of respiratory virus PCR tests according to each level of social distancing.~~ The more stringent the level of social distancing, the higher was the proportion of negative respiratory virus PCR test results (*P* < 0.001 using the Kruskal-Wallis test; the results of post hoc comparisons using Mann-Whitney *U* test with Bonferroni correction are presented in Table 2). The number of respiratory virus PCR tests was significantly different between the periods with and without social distancing – adjusted *P* value is <0.001 when comparing the “no social distancing group” and the level 1, 2, and 3 social distancing group, respectively (Table 2). However, when the three social distancing groups were compared with each other in the number of respiratory virus PCR tests, there was no significant difference (Table 2).

**Fig 2. Mean proportion of negative respiratory virus PCR test results according to the level of social distancing (between the 1st week of 2015 and the 42nd week of 2020; a total of 303 weeks) and the mean number of respiratory virus tests according to the level of social distancing (between the 23rd week of 2017 and the 39th week of 2020; a total of 176 weeks). The horizontal line represents the mean value, and the hollow circle represents the value for each week.**

PCR, Polymerase chain reaction

*To) The 3^rd^ paragraph of the results*

The more stringent the level of social distancing, the higher was the proportion of negative respiratory virus PCR test results as shown in the left graph of Figure 2 (*P* < 0.001 using the Kruskal-Wallis test; the results of post hoc comparisons using Mann-Whitney *U* test with Bonferroni correction are presented in Table 2). However, this dose-response relationship was not observed between the level of social distancing and the number of respiratory virus PCR test as shown in the right graph of Figure 2, although the number was significantly different between the periods with and without social distancing – adjusted *P* value is <0.001 when comparing the “no social distancing group” and the level 1, 2, and 3 social distancing group, respectively (Table 2).

**Fig 2. Mean proportion of negative respiratory virus PCR test results according to the level of social distancing (between the 1st week of 2015 and the 42nd week of 2020; a total of 303 weeks) and the mean number of respiratory virus tests according to the level of social distancing (between the 23rd week of 2017 and the 39th week of 2020; a total of 176 weeks).** The horizontal line represents the mean value, and the hollow circle represents the value for each week. PCR, Polymerase chain reaction

*From) Table 2*

**Table 2. Post hoc comparisons of the proportion of negative respiratory virus PCR test results and the number of respiratory virus PCR tests according to the levels of social distancing (SD) (levels 0, 1, 2, and 3) using the Mann-Whitney *U* test with Bonferroni correction.**

| **Proportion of negative PCR test results** | | | | **No. of PCR tests** | | | |
| --- | --- | --- | --- | --- | --- | --- | --- |
| **~~Group~~** | **Comparison** | ***P* value** | **Adjusted *P* value** | **~~Group~~** | **Comparison** | ***P* value** | **Adjusted *P* value** |
| ~~1~~ | SD level 0 vs. 1 | 0.051 | 0.306 | ~~1~~ | SD level 0 vs. 1 | <0.001 | **<0.001** |
| ~~2~~ | SD level 0 vs. 2 | <0.001 | **<0.001** | ~~2~~ | SD level 0 vs. 2 | <0.001 | **<0.001** |
| ~~3~~ | SD level 0 vs. 3 | <0.001 | **<0.001** | ~~3~~ | SD level 0 vs. 3 | <0.001 | **<0.001** |
| ~~4~~ | SD level 1 vs. 2 | 0.004 | **0.024** | ~~4~~ | SD level 1 vs. 2 | 0.13 | 0.78 |
| ~~5~~ | SD level 1 vs. 3 | <0.001 | **<0.001** | ~~5~~ | SD level 1 vs. 3 | 0.255 | 1.53 |
| ~~6~~ | SD level 2 vs. 3 | 0.083 | 0.498 | ~~6~~ | SD level 2 vs. 3 | 0.197 | 1.182 |

*To) Table 2*

**Table 2. Post hoc comparisons of the proportion of negative respiratory virus PCR test results and the number of respiratory virus PCR tests according to the levels of social distancing (SD) (levels 0, 1, 2, and 3) using the Mann-Whitney *U* test with Bonferroni correction.**

**NOTE.** SD level 0 presents refers to the years 2015-2019 when SD was not applied.

| **Proportion of negative PCR test results** | | | **No. of PCR tests** | | | |
| --- | --- | --- | --- | --- | --- | --- |
| **Comparison** | ***P* value** | **Adjusted *P* value** | **Comparison** | ***P* value** | **Adjusted *P* value** | |
| SD level 0 vs. 1 | 0.051 | 0.306 | SD level 0 vs. 1 | <0.001 | **<0.001** |  |
| SD level 0 vs. 2 | <0.001 | **<0.001** | SD level 0 vs. 2 | <0.001 | **<0.001** |  |
| SD level 0 vs. 3 | <0.001 | **<0.001** | SD level 0 vs. 3 | <0.001 | **<0.001** |  |
| SD level 1 vs. 2 | 0.004 | **0.024** | SD level 1 vs. 2 | 0.13 | 0.78 |  |
| SD level 1 vs. 3 | <0.001 | **<0.001** | SD level 1 vs. 3 | 0.255 | 1.53 |  |
| SD level 2 vs. 3 | 0.083 | 0.498 | SD level 2 vs. 3 | 0.197 | 1.182 |  |

*From) The 4^th^ paragraph of the results*

~~Figure 3 presents the curves of the weekly number/weekly positive rates for each of the eight respiratory viruses. In Table 3, the mean weekly positive rates of respiratory viruses were compared between the social distancing period and the corresponding periods of 2015–2019. Additionally, the same comparisons were conducted according to the three levels of social distancing.~~ Each of PIV, IFV, COV, and MPV is rarely observed in the proportion graphs and number graphs in 2020, when social distancing was implemented, unlike 2015-2019 (B-1, B-2, D-1, D-2, E-1, E-2, H-1, and H-2 of Fig 3). In Table 3, the mean positive rates for PIV, IFV, COV, and MPV during the social distancing period were significantly lower than those during the same period in 2015–2019, when social distancing was not implemented (0.1% vs. 9.3%, *P* <0.001; 0.1% vs. 7.2%, *P* <0.001; 0.4% vs. 2.3%, *P* <0.001; and 0.2% vs. 5.3%, *P* <0.001, respectively). This trend was maintained during each level of social distancing (Table 3). ~~The mean positive rate for RSV during level 2 social distancing was lower than that during the same period in 2015–2019 (0.3% vs. 1.8%,~~ *~~P~~* ~~= 0.004). However, considering both Table 3 and Figure 3 (C-1 and C-2),~~ social distancing was implemented outside the general RSV outbreak period; thus, it was difficult to observe the effects of social distancing with respect to RSV positivity in this dataset. The number of ADV and BOV each appeared to decrease in 2020 compared to 2015-2019 period in the number graphs (A-2 and G-2 of Fig 3), ~~but there was no significant difference between the 2015-2019 period and 2020 in the proportion graphs~~ (A-1 and G-1 of Fig 3). In Table 3, there were no differences in mean positive rates of ADV and BOV between the social distancing period and the same period in 2015–2019 ~~(6.2% vs. 6.2%,~~ *~~P~~* ~~= 0.906 and 2.6% vs. 3.1%,~~ *~~P~~* ~~= 0.432, respectively)~~. In the proportion graph, RV/EV decreased during the level 2-3 social distancing period in 2020 compared to 2015-2019, but increased in the level 1 social distancing period compared to 2015-2019 (F-1 of Fig 3). ~~In the number graph of RV/EV, the number decreased during the level 2-3 social distancing period, but increased to a level similar to that of 2015-2019 during the level 1 social distancing period (F-2 of Fig 3).~~ The mean positive rate of RV/EV during level 3 social distancing was lower than that in the same period in 2015–2019 (8.5% vs. 19.0%, *P* <0.001); however, during level 1 social distancing, the mean positive rate was higher than that in the same period in 2015–2019 (38.3% vs. 19.4%, *P* <0.001) (Table 3). ~~Except for RV/EV of the level 1 social distancing period, weekly numbers of each of seven respiratory viruses during the periods of social distancing were generally lower than those without social distancing (Fig 3), because less than half of the respiratory virus PCR tests were conducted during the former periods compared to the latter periods.~~ Graphs of weekly positive rates for the eight respiratory viruses from the university hospital dataset are presented in S2 Fig.

**Fig 3. Weekly number and positive rates of respiratory viruses in South Korea between the 1st week of 2015 and the 42nd week of 2020**

A-1. Proportion of ~~Adenovirus (ADV)~~; A-2. Number of ~~ADV~~; B-1. Proportion of ~~Parainfluenza virus (PIV)~~; B-2. Number of ~~PIV~~; C-1. Proportion of ~~Respiratory syncytial virus (RSV)~~; C-2. Number of ~~RSV~~; D-1. Proportion of ~~Influenza (IFV)~~; D-2. Number of ~~IFV~~; E-1. Proportion of ~~Human coronavirus (COV)~~; E-2. Number of ~~COV~~; F-1. Proportion of ~~Rhinovirus/enterovirus (RV/EV)~~; F-2. Number of ~~RV/EV~~; G-1. Proportion of Human bocavirus (BOV); G-2. Number of BOV; H-1. Proportion of ~~Human metapneumovirus (MPV)~~; H-2. Number of ~~MPV.~~ Each of gray squares represents the periods of level 3, level 1, and level 2 social distancing.

*To) The 4^th^ paragraph of the results*

Each of PIV, IFV, COV, and MPV is rarely observed in the proportion graphs and number graphs in 2020, when social distancing was implemented, unlike 2015-2019 (A-1, A-2, B-1, B-2, C-1, C-2, D-1, and D-2 of Figure 3). In Table 3, the mean positive rates for PIV, IFV, COV, and MPV during the social distancing period were significantly lower than those during the same period in 2015–2019, when social distancing was not implemented (0.1% vs. 9.3%, *P* <0.001; 0.1% vs. 7.2%, *P* <0.001; 0.4% vs. 2.3%, *P* <0.001; and 0.2% vs. 5.3%, *P* <0.001, respectively). This trend was maintained during each level of social distancing (Table 3). It was difficult to observe the effects of social distancing with respect to RSV positivity in this dataset, because social distancing was implemented outside the general RSV outbreak period (E-1 and E-2 of Fig 3, and Table 3). Although the number of ADV and BOV each appeared to decrease in 2020 compared to 2015-2019 period, there were no differences in positive rates of ADV and BOV between the social distancing period and the corresponding period in 2015–2019 (F-1, F-2, G-1, and G-2 of Fig 3, and Table 3). The proportion of RV/EV decreased during the level 2-3 social distancing period in 2020 compared to 2015-2019, but increased in the level 1 social distancing period (H-1 and H-2 of Fig 3, and Table 3). Graphs of weekly positive rates for the eight respiratory viruses from the university hospital dataset are presented in S2 Fig.

**Fig 3. Weekly number and positive rates of respiratory viruses in South Korea between the 1st week of 2015 and the 42nd week of 2020**

A-1. Proportion of Parainfluenza virus (PIV); A-2. Number of PIV; B-1. Proportion of Influenza virus (IFV); B-2. Number of IFV; C-1. Proportion of Human coronavirus (COV); C-2. Number of COV; D-1. Proportion of Human metapneumovirus (MPV); D-2. Number of MPV; E-1. Proportion of Respiratory syncytial virus (RSV); E-2. Number of RSV; F-1. Proportion of Adenovirus (ADV); F-2. Number of ADV; G-1. Proportion of Human bocavirus (BOV); G-2. Number of BOV; H-1. Proportion of Rhinovirus/enterovirus (RV/EV); H-2. Number of RV/EV. Each of gray squares represents the periods of level 3, level 1, and level 2 social distancing.

**Discussion, 2nd para, the authors claimed "This is the first time that the effect of social distancing on the outbreaks of common respiratory viruses has been confirmed using national data from a country where social distancing is well implemented." I wonder if this is the case, as similar study was published in other cities, e.g. The Lancet Public Health VOLUME 5, ISSUE 5, E279-E288, MAY 01, 2020 (reference 11 as the authors indicated); and the authors themselves also indicated a similar study as in reference 5.**

*Answer) As you pointed out, similar research data have already been reported, so the sentence has been deleted.*

This study shows that social distancing prevented outbreaks of common respiratory viruses, and this effect was proportional to the level of social distancing. ~~This is the first time that the effect of social distancing on the outbreaks of common respiratory viruses has been confirmed using national data from a country where social distancing is well implemented.~~ The impact of diverse non-pharmaceutical interventions against COVID-19 has been reported during this pandemic [5]. Additionally, the reduction of the influenza epidemic due to social distancing has already been reported in several studies [11–13]. Common respiratory viruses are transmitted in a manner similar to SARS-CoV-2 and influenza virus. Thus, social distancing is expected to have some effect in terms of suppressing the spread of common respiratory viruses. The results of this study clearly show how extensive social distancing helps prevent the spread of various respiratory viruses, even without the use of antiviral drugs or vaccines. Thus, extensive social distancing may be one of the most effective methods to control a pandemic of similar respiratory viruses. In contrast, the proportion of negative respiratory virus PCR test results may be a surrogate marker to identify whether social distancing is being properly implemented. In May 2020, the media focused on the issue of improper implementation of social distancing (good weather for outdoor activities and various holidays). Therefore, the proportion of negative respiratory virus PCR test results decreased to 60% even before the discontinuation of social distancing (Fig 1). Furthermore, the impact of social distancing on the occurrence of common respiratory virus-associated acute illnesses such as pneumonia, acute exacerbation of chronic obstructive lung disease or asthma, and cardiovascular or cerebral vascular events need to be investigated based on the status of the current pandemic.

***Thank you for your thoughtful comments!***
